# A prediction model for thrombocytopenia after neurosurgery: a retrospective study

**DOI:** 10.7717/peerj.21094

**Published:** 2026-04-17

**Authors:** Chenlu Zhang, Shoumei Jia, Yuli Zang, Xuandong Jiang

**Affiliations:** 1 Affiliated Dongyang Hospital of Wenzhou Medical University, Jinhua, Zhejiang Province, China; 2 School of Nursing, Fudan University, Shanghai, China; 3 School of Medicine, Hangzhou City University, Hangzhou, Zhejiang Province, China; 4 Intensive Care Unit, Affiliated Dongyang Hospital of Wenzhou Medical University, Jinhua, Zhejiang Province, China

**Keywords:** Postoperative thrombocytopenia, Prediction, Logistic regression, Neurosurgery

## Abstract

**Objective:**

Thrombocytopenia frequently occurs after major surgery and is linked to negative clinical outcomes. This study aimed to predict thrombocytopenia occurrence in patients following neurosurgery using a logistic regression model.

**Methods:**

We retrospectively analyzed 1,109 postoperative patients who had neurosurgery between January 2010 and December 2020 at the Dong Yang People’s Hospital. We obtained medical records, encompassing demographic details, clinical outcomes, and laboratory results, from the hospital’s database. The variables included in the model were identified using least absolute shrinkage and selection operator (LASSO) regression. Data from the research participants were split into training and test sets. The training dataset was employed for constructing a logistic regression model, while the test dataset was utilized to test the model’s performance. Additionally, we performed subgroup analysis by etiology.

**Results:**

Among 1,109 patients, 103 developed thrombocytopenia (9.3%). Patients with thrombocytopenia had a longer hospital stay and mechanical ventilation time than those without. The eight predictive variables selected through LASSO regression for modeling were: hypertension, Sepsis-related Organ Failure Assessment (SOFA) score, serum total bilirubin and albumin, International Normalized Ratio, thrombocytocrit, first measured systolic blood pressure and use of vasoactive drugs. The logistic regression model demonstrated satisfactory discriminative ability, showing an area under the curve of 0.916 (95% confidence interval [0.886–0.947]) for the training set and 0.883 [0.817–0.950] for the test set, coupled with high specificity (>0.9) but low recall (0.414 training, 0.364 test) at a cutoff value of 0.5. Calibration curves also indicated good predictive accuracy for both sets. Subgroup analysis revealed that the model for acute brain injury achieved a robust optimism-corrected AUC of 0.905, with precision improving from 0.545 to 0.664.

**Conclusion:**

The logistic regression models from this study have the potential to predict thrombocytopenia following neurosurgery and can serve as clinical decision-support tools for early intervention.

## Introduction

Postoperative thrombocytopenia is a frequent and serious complication, observed in a substantial proportion of patients in the Intensive Care Unit (ICU) and general surgical settings (Nagrebetsky et al., 2019). The degree of thrombocytopenia may be associated with the type of surgery (Skeith et al., 2020). Compared with patients without postoperative thrombocytopenia, those who develop it are reported to have higher in-hospital mortality and morbidity, including post-operative infection, post-operative stroke, acute kidney injury, and extended hospital and ICU stays after cardiac surgery (Griffin et al., 2020; Cao et al., 2022; Tang & Shao, 2023). Within the ICU setting, thrombocytopenia typically signifies profound systemic dysfunction and physiological decompensation, rather than an isolated hematologic issue. It is consequently associated with complications including but not limited to bleeding, a greater need for blood product transfusions, and increased mortality (Zarychanski & Houston, 2017). A prospective multicenter study demonstrated that thrombocytopenia at ICU admission serves as an independent predictor of 90-day mortality (Thiolliere et al., 2013).

While postoperative thrombocytopenia is a prevalent complication across various surgical specialties, its etiology, clinical course, and associated risks are uniquely heightened in the neurosurgical population. Firstly, normal hemostatic function is crucial for neurosurgical procedures, as even minor bleeding can have severe, catastrophic consequences for patients (Gerlach et al., 2004). Neurosurgical procedures and acute cerebral injuries—such as traumatic brain injury (TBI) or tumor resection—often trigger unique and severe hemostatic derangements. Unlike other surgeries, the brain parenchyma is rich in tissue factor (TF), and its injury can lead to a massive release of TF into the systemic circulation, rapidly initiating the extrinsic coagulation cascade (Salem et al., 2019). This often results in a profound state of consumptive coagulopathy and platelet depletion, which has been identified as a strong predictor of poor clinical outcomes in postoperative neurosurgical cohorts (Sharma et al., 2023). Secondly, the clinical consequences of thrombocytopenia are catastrophic in the neurosurgical context. Even minor drops in platelet count significantly increase the risk of secondary intracranial hemorrhage (ICH), which can lead to rapid neurological deterioration and death. Consequently, the platelet transfusion threshold and the demand for early risk identification in neurosurgery are significantly higher than in other specialties (Salem et al., 2019).

Thrombocytopenia is already recognized as a strong risk factor for hematomas after neurosurgical procedures (Li, Glor & Jones, 2017). For example, a retrospective case review by Chan, Mann & Chan (1989) showed that a postoperative platelet count <100,000/µl had a significant association with postoperative hematoma, especially when platelet transfusion was ineffective. Furthermore, thrombocytopenia has been reported to significantly increase short-term mortality after surgery for traumatic brain injury (Xu et al., 2023), and patients who recovered from thrombocytopenia during their neurological ICU stay had a decreased rate of in-hospital mortality compared to those with continuous thrombocytopenia (Zhou et al., 2020).

Existing prediction models for postoperative thrombocytopenia are predominantly derived from specific cohorts such as cardiac surgery (Li et al., 2023; Zhao et al., 2024). No research has been conducted on developing models explicitly designed to predict thrombocytopenia in patients after neurosurgery. Logistic regression is the traditional method used to build a prediction model and is now extensively applied in medical studies for outcome forecasting (Huang, Chen & Miao, 2022). Thus, in this study, we aimed to construct a logistic regression model for predicting thrombocytopenia following neurosurgery.

## Materials and Methods

This retrospective study included 1,109 adult patients (≥18 years) who underwent invasive neurosurgical interventions between January 2010 and December 2020 at the Dong Yang People’s Hospital. The cohort encompassed a full spectrum of procedures, ranging from major craniotomies to minor operative interventions such as burr-hole drainage, cranioplasty, and external ventricular drainage (EVD). Data from the research participants were split into training and test sets. We used the training set to construct a logistic regression model and subsequently tested the model using the test set. In addition, we also performed subgroup analysis by etiology. Exclusion criteria included a history of cirrhosis, hematological malignancy, preoperative thrombocytopenia, heparin-induced thrombocytopenia (HIT), thrombotic thrombocytopenic purpura (TTP), and previous splenectomy. This study received approval from the Ethics Committee of Dong Yang People’s Hospital (DRY-2022-YX-155). Given the observational and retrospective nature of the study, requirement informed consent was waived. Personal information was removed prior to the analysis, and data were analyzed anonymously.

### Data collection

Medical records of included patients were collected from the Dong Yang People’s Hospital database. The collected data encompassed a range of variables including: demographic information; comorbidities, initial clinical status and severity scores (*e.g*., Acute Physiology and Chronic Health Evaluation II (APACHE-II) score, Glasgow Coma Scale (GCS), and Sepsis-related Organ Failure Assessment (SOFA) score); preoperative diagnosis categories and surgical site; laboratory parameters; and relevant clinical outcomes. To ensure the model’s utility for early clinical decision support, all predictor variables were collected relative to the time of surgical completion and ICU admission. In our clinical practice, all neurosurgical patients were transferred directly to the ICU immediately following surgery for standardized postoperative monitoring. The timing of data collection was strictly defined as follows: (i) Baseline characteristics and comorbidities (*e.g*., hypertension) were recorded upon hospital admission. (ii) Laboratory values (*e.g*., International Normalized Ratio (INR), serum albumin, and total bilirubin) were obtained from the first blood draw immediately upon ICU admission. (iii) Postoperative physiological parameters (*e.g*., heart rate, respiratory rate, body temperature, and systolic/diastolic blood pressure), including initial, maximum, minimum, and mean values, along with clinical severity scores and medication use, were collected or calculated within the first 24 h of ICU stay. The primary outcome was the development of thrombocytopenia, defined as a platelet count < 100 × 10^9^/L (Ben et al., 2003; Thiolliere et al., 2013), at any time during the ICU stay.

### Data processing

#### Selection of independent variables

**Figure 1:**
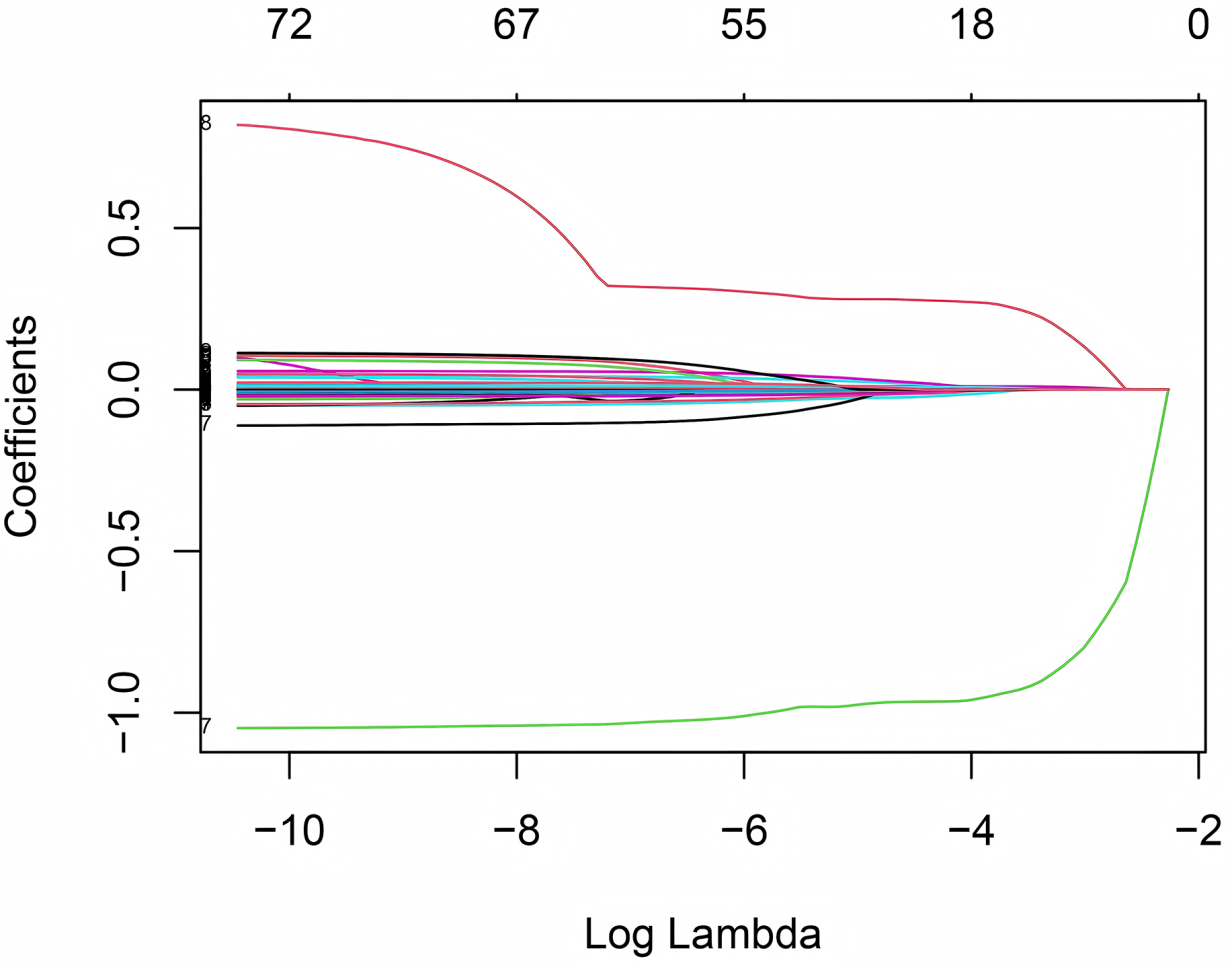
Variable selection using the least absolute shrinkage and selection operator (LASSO) regression model. Each curve represents the coefficient path of a predictor variable as the penalty parameter (log(λ)) increases.

**Figure 2:**
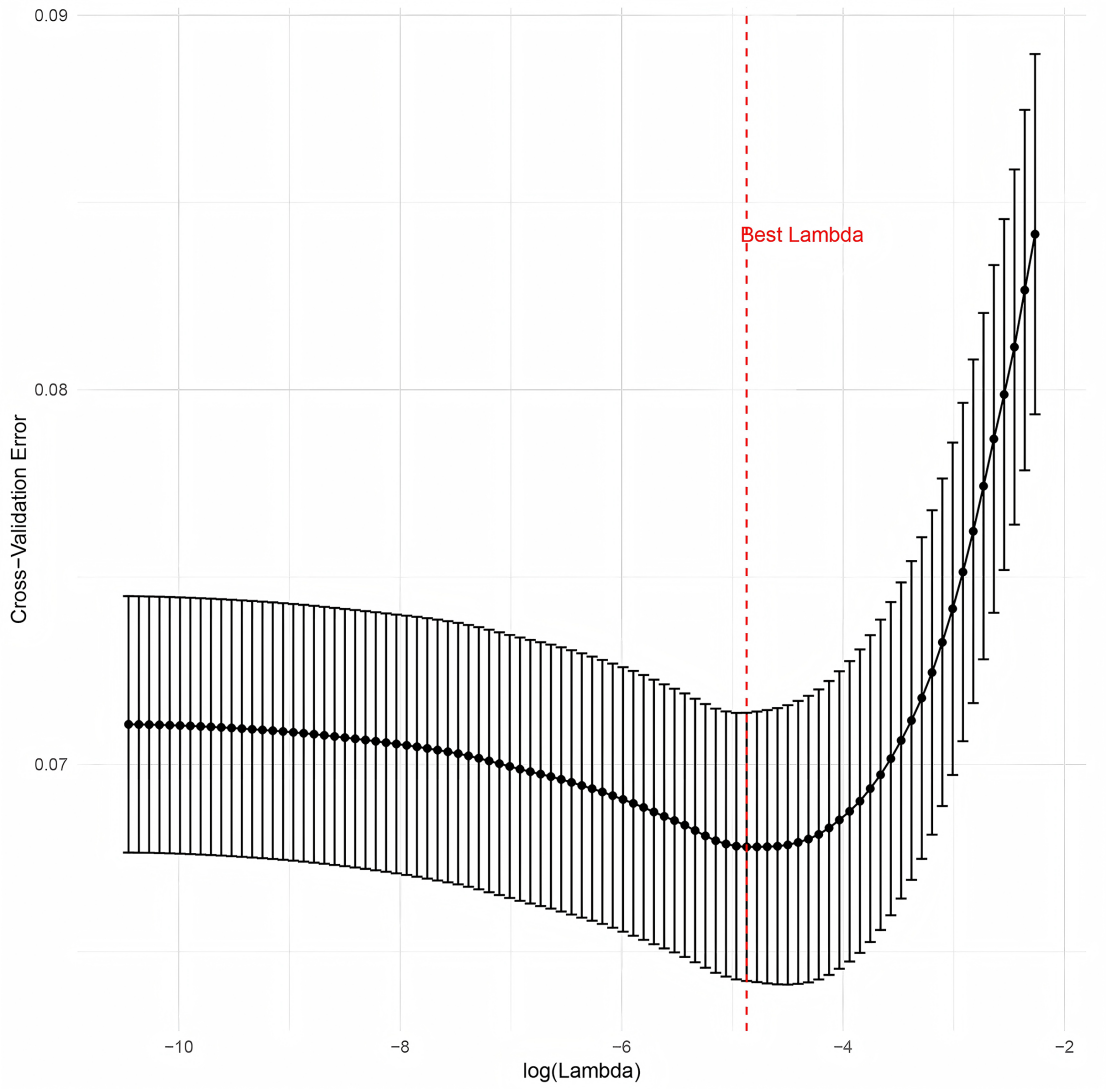
Cross-validation curve for tuning parameter (λ) selection in the LASSO regression. The mean cross-validation error (y-axis) is plotted against log(λ) (x-axis).

**Figure 3:**
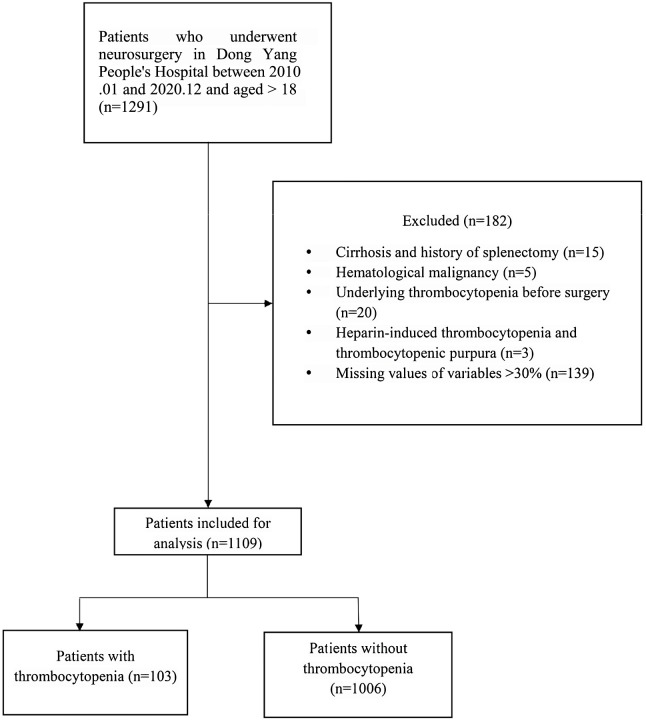
Flow chart of the study.

A total of 101 variables related to patient information were collected from the database. The initial screening eliminated seventeen variables. These excluded variables comprised: (i) those representing longer-term patient outcomes (*e.g*., length of stay, mortality) to prevent target leakage; (ii) those providing redundant clinical information (specifically, repeated measures of platelet count and renal function) to mitigate multicollinearity; and (iii) two variables with no variation. From the remaining 84 variables, three exhibiting strong correlations (Pearson’s r > 0.8) with other variables were excluded, leaving 81 independent variables that were subsequently entered into the least absolute shrinkage and selection operator (LASSO) regression. To conduct LASSO regression, we utilized the GLMNET package in R (Li, Lu & Yin, 2022). After performing the LASSO regression, eight variables were ultimately chosen to build the logistic regression prediction model: hypertension, SOFA score, thrombocytocrit, INR, serum total bilirubin, serum albumin, first measured systolic blood pressure, and use of vasoactive drugs. Figure 1 shows the LASSO coefficient path, and Fig. 2 displays the cross-validation error *vs* log(λ). A flow chart of the study is shown in Fig. 3.

### Handling of outliers and missing values

The upper quartile plus 1.5 times the interquartile range (IQR) was considered the upper bound, and the lower quantile minus 1.5 times the IQR was considered the lower bound. Values greater than the upper bound or smaller than the lower bound were considered outliers and replaced by the upper or lower bound. Variables with missing values exceeding 30% were deleted. Variables with <30% missing values were processed with multiple imputation in the R language with the MICE package (Zhang, 2016). We performed five imputations and randomly selected one for the final analysis.

#### Model establishment

Patient data were separated into training and test sets with a proportion of 7:3. We constructed a logistic regression model using the training set. Multicollinearity among the predictor variables was examined with R. The variance inflation factor (VIF) values for all variables were under 10, suggesting no issues with multicollinearity in the model. A summary of algorithms and modeling approaches employed in this study is provided in Table S1.

#### Model testing and evaluation

The model was tested with the test set and evaluated using the receiver operating characteristic (ROC) curve and calibration curve. The area under the ROC curve (AUC) with its 95% confidence interval (CI), along with key classification metrics (recall, specificity, precision, accuracy, and F1-score) based on a 0.5 cutoff value (Jiang et al., 2022a) were calculated. In addition, we constructed a nomogram using the test set.

#### Subgroup analysis

For the subgroup analysis, patients were categorized into two primary groups based on the nature of their admission: the ‘Acute Injury’ subgroup, comprising those with intracranial hemorrhage (ICH) and traumatic brain injury (TBI), and the ‘Elective Surgery’ subgroup, which included patients with intracranial tumors and unruptured cerebrovascular diseases. We utilized LASSO regression on the entire dataset of the acute injury subgroup to select predictor variables. Due to the limited number of events in the subgroups (97 cases in the acute group), we employed 1,000-iteration Bootstrap resamplings instead of a simple train-test split to ensure model robustness and to maximize the utility of the available data. Model performance was evaluated using ROC curves and the calculation of recall, specificity, precision, accuracy, and F1-score at a cutoff value of 0.5. In the elective surgery subgroup, only one patient developed thrombocytopenia (0.9%, 1/109). Given this extreme paucity of events, independent variable selection *via* LASSO or regression modeling was statistically unfeasible. Consequently, we validated the primary model’s performance in this subgroup by calculating its specificity and negative predictive value (NPV).

#### Statistical analysis

The *CBCgrps* package in R was used to analyze descriptive statistics (Jiang et al., 2022b). Skewed data were described with the median (P75, P25), normally distributed data were described with the mean and standard deviation, categorical variables were described with percentages. The differences between groups for these three types of data were analyzed with the Mann–Whitney U test, independent two-sample t-test, and Chi-square test, respectively. A *P* value < 0.05 was considered statistically significant.

## Results

### Comparison of baseline characteristics and clinical outcomes

This study included 1,109 patients, 103 of whom developed thrombocytopenia, with an incidence rate of approximately 9.3%. Table 1 displays a comparison of the patient’s baseline characteristics and clinical outcomes. Patients with thrombocytopenia were significantly older (60.3 ± 14.6 years) than those without (55.8 ± 15.2 years, *P* = 0.004). No significant differences were observed in smoking history, sex, alcohol drinking history, chronic kidney disease, or diabetes prevalence between the two groups. Hypertension was more prevalent, and the first measured systolic blood pressure was higher among patients without than those with thrombocytopenia (*P* < 0.001). The thrombocytopenia group had a significantly elevated APACHE-II score, SOFA score, INR, total bilirubin level, D-dimer level, and aspartate aminotransferase level; lower GCS, serum albumin and thrombocytocrit, and prolonged prothrombin and activated partial thromboplastin times. The distribution of preoperative diagnosis differed significantly between groups. In addition, a higher percentage of patients with thrombocytopenia used vasoactive drugs than those without (*P* < 0.001). The difference in hospital mortality between the two groups was not significant, but the thrombocytopenia group experienced longer ICU and hospital stays and durations of mechanical ventilation (*P* < 0.001).

**Table 1:** Comparison of baseline characteristics among those with and without thrombocytopenia.

	Thrombocytopenia (*n* = 103)	No-thrombocytopenia (*n* = 1,006)	Total (*n* = 1,109)	*P*-value
**Basic information**				
Age (years)	60.3 ± 14.6	55.8 ± 15.2	56.2 ± 15.2	0.004
Male (*n* (%))	65 (63.1)	617 (61.3)	682 (61.5)	0.805
Alcohol drinking (*n* (%))	38 (36.9)	405 (40.3)	443 (39.9)	0.576
Smoking (n (%))	36 (35)	347 (34.5)	383 (34.5)	1
APACHE-II	22.1 ± 5.5	17.5 ± 6	17.9 ± 6.1	<0.001
SOFA score	7 ± 2.1	4.6 ± 2.4	4.8 ± 2.5	<0.001
Antiplatelet drug used (*n* (%))	1 (1)	3 (0.3)	4 (0.4)	0.323
Vasoactive drug used *(n* (%))	32 (31.1)	140 (13.9)	172 (15.5)	<0.001
First measured systolic blood pressure (mmHg)	145 ± 30.4	159.7 ± 30.1	158.3 ± 30.4	<0.001
Preoperative diagnosis (*n* (%))				<0.001
Intracranial hemorrhage	30 (29.1)	525 (52.2)	555 (50.0)	
Traumatic brain injury	67 (65.0)	349 (34.7)	416 (37.5)	
Intracranial tumors	0 (0.0)	43 (4.3)	43 (3.9)	
Unruptured cerebrovascular diseases	1 (1.0)	65 (6.5)	66 (6.0)	
Other	5 (4.9)	24 (2.4)	29 (2.6)	
Surgical site (*n* (%))				0.074
Supratentorial	78 (75.7)	645 (64.1)	723 (65.2)	
Infratentorial	1 (1.0)	7 (0.7)	8 (0.7)	
Skull base	18 (17.5)	222 (22.1)	240 (21.6)	
Other	6 (5.8)	132 (13.1)	138 (12.4)	
GCS	8.0 (5.5, 10.0)	9.0 (6.0, 12.0)	9.0 (6.0, 12.0)	0.002
**Comorbidities**				
CKD (n (%))	0 (0)	6 (0.6)	6 (0.5)	1
Diabetes (n (%))	7 (6.8)	72 (7.2)	79 (7.1)	1
Hypertension (n (%))	25 (24.3)	491 (48.8)	516 (46.5)	< 0.001
**Postoperative complications**				
Sepsis (*n* (%))	75 (72.8)	576 (57.3)	651 (58.7)	0.003
Deep vein thrombosis (*n* (%))	18 (17.5)	97 (9.6)	115 (10.4)	0.021
Gastrointestinal bleeding (*n* (%))	7 (6.8)	44 (4.4)	51 (4.6)	0.384
**Initial biochemical indicators**				
White blood cell (×10^9^/L)	10.3 (7.6, 13.3)	10.8 (8.5, 13.3)	10.7 (8.5, 13.3)	0.233
Neutrophil count (×10^9^/L)	8.9 (6.4, 11.3)	9 (7, 11.4)	9 (7, 11.4)	0.75
Lymphocyte count (×10^9^/L)	0.8 (0.6, 1)	1 (0.7, 1.5)	1 (0.7, 1.4)	<0.001
Red blood cell (×10^12^/L)	3.3 ± 0.6	3.8 ± 0.6	3.7 ± 0.6	<0.001
Hematocrit (L/L)	0.3 ± 0.1	0.3 ± 0	0.3 ± 0.1	<0.001
pH	7.4 ± 0.1	7.4 ± 0.1	7.4 ± 0.1	0.075
Platelet count (×10^9^/L)	99 (82.5, 114)	170 (138.2, 206)	164 (130, 203)	<0.001
Thrombocytocrit (%)	0.1 ± 0	0.2 ± 0.1	0.2 ± 0.1	<0.001
Serum sodium (mmol/L)	140.7 ± 3.4	140.7 ± 3.3	140.7 ± 3.3	0.877
Serum calcium (mmol/L)	2 ± 0.1	2.1 ± 0.1	2.1 ± 0.1	<0.001
Serum bicarbonate (mmol/L)	22.2 ± 2.5	22.5 ± 2.4	22.5 ± 2.4	0.175
Serum lactate (mmol/L)	2.1 (1.5, 3.5)	1.8 (1.2, 2.9)	1.9 (1.3, 2.9)	0.001
Serum total bilirubin (µmol/L)	18.2 (13.4, 26.4)	13.3 (10.1, 17.9)	13.7 (10.3, 18.3)	<0.001
International normalized ratio	1.2 (1.1, 1.3)	1.1 (1, 1.2)	1.1 (1, 1.2)	<0.001
Prothrombin time (s)	15.3 (14.6, 16.4)	14.2 (13.6, 14.9)	14.3 (13.6, 15.1)	<0.001
Activated partial thromboplastin time (s)	38.6 (35, 42.3)	36.6 (34.1, 39.6)	36.7 (34.2, 39.8)	<0.001
D-dimer (µg/L)	7.7 (3.4, 16)	3.1 (1.4, 7.6)	3.4 (1.4, 8.5)	<0.001
Serum albumin (g/L)	31.3 ± 4.4	34.3 ± 4.1	34.1 ± 4.2	<0.001
Serum creatinine (µmol/L)	56 (46.5, 70.5)	58 (47, 69)	58 (47, 70)	0.859
Serum urea (mmol/L)	4.7 (3.8, 6.3)	4.8 (3.6, 6.2)	4.8 (3.7, 6.2)	0.68
Alanine aminotransferase (U/L)	19 (13, 27)	16 (12, 23)	16 (12, 24)	0.02
Aspartate aminotransferase (U/L)	31 (25, 42)	24 (19, 35)	25 (20, 35)	<0.001
**Outcome**				
Surgery time (hours)	3.4 ± 1.4	3.2 ± 1.3	3.2 ± 1.3	0.14
ICU length of stay (days)	10.4 (6.6, 16.5)	4.3 (1.9, 9.8)	4.7 (2.2, 10.6)	<0.001
Mechanical ventilation time (days)	5.5 (2.5, 10.7)	1.6 (0.7, 5.3)	1.8 (0.7, 5.9)	<0.001
Hospital length of stay (days)	23 (18, 31)	19 (15, 25)	19 (15, 25)	<0.001
Hospital mortality (*n* (%))	4 (3.9)	24 (2.4)	28 (2.5)	0.321

**Note:**

Continuous variables are described by means and quarterbacks. Categories variables are analyzed by $\chi^2$ test and continuous variables are analyzed by Wilcoxon rank sum test. APACHE, Acute physiology and chronic health evaluation; GCS, glasgow coma scale; ICU, intensive care unit; CKD, chronic kidney disease; SOFA, sepsis-related organ failure assessment.

Table 2 displays the baseline data of non-survivors and survivors. Of the 1,109 patients, 28 (2.5%) died. Survivors were younger (56 ± 15.2 *vs* 62.9 ± 14.6 years, *P* = 0.02), had a lower APACHE-II score (17.8 ± 6.1 *vs* 21 ± 6.2, *P* = 0.012) and a higher red blood cell count (3.7 ± 0.6 *vs* 3.4 ± 0.6, *P* = 0.022) than non-survivors. Furthermore, non-survivors experienced significantly longer ICU stays (*P* = 0.003), extended mechanical ventilation times (*P* < 0.001), and increased incidence of sepsis than survivors (*P* = 0.018).

**Table 2:** Comparison of baseline characteristics between survivors and non-survivors.

	Survivors (*n* = 1,081)	Non-survivors (*n* = 28)	Total (*n* = 1,109)	*P*-value
**Basic information**				
Age (years)	56 ± 15.2	62.9 ± 14.6	56.2 ± 15.2	0.02
Male (*n* (%))	665 (61.5)	17 (60.7)	682 (61.5)	1
Alcohol Drinking (*n* (%))	433 (40.1)	10 (35.7)	443 (39.9)	0.789
Smoking (*n* (%))	379 (35.1)	4 (14.3)	383 (34.5)	0.037
APACHE-II	17.8 ± 6.1	21 ± 6.2	17.9 ± 6.1	0.012
SOFA score	4.8 ± 2.4	5.6 ± 2.6	4.8 ± 2.5	0.131
Vasoactive drug used (*n* (%))	164 (15.2)	8 (28.6)	172 (15.5)	0.063
First measured systolic blood pressure (mmHg)	158.6 ± 30.3	146.8 ± 31.7	158.3 ± 30.4	0.061
Antiplatelet drug used (*n* (%))	4 (0.4)	0 (0)	4 (0.4)	1
**Comorbidities**				
CKD (*n* (%))	5 (0.5)	1 (3.6)	6 (0.5)	0.143
Diabetes (*n* (%))	76 (7)	3 (10.7)	79 (7.1)	0.444
Hypertension (*n* (%))	498 (46.1)	18 (64.3)	516 (46.5)	0.086
**Postoperative complications**				
Sepsis (*n* (%))	628 (58.1)	23 (82.1)	651 (58.7)	0.018
**Initial biochemical indicators**				
White blood cell (×10^9^/L)	10.8 (8.5, 13.3)	9.5 (7.5, 12.5)	10.7 (8.5, 13.3)	0.192
Neutrophil count (×10^9^/L)	9 (7, 11.4)	8 (6, 10.7)	9 (7, 11.4)	0.268
Lymphocyte count (×10^9^/L)	1 (0.7, 1.4)	0.9 (0.7, 1.1)	1 (0.7, 1.4)	0.141
Red blood cell (×10^12^/L)	3.7 ± 0.6	3.4 ± 0.6	3.7 ± 0.6	0.022
pH	7.4 ± 0.1	7.4 ± 0.1	7.4 ± 0.1	0.284
Platelet count (×10^9^/L)	164 (130, 203)	163 (123, 197.2)	164 (130, 203)	0.976
Serum bicarbonate (mmol/L)	22.5 ± 2.4	23 ± 2	22.5 ± 2.4	0.161
Serum lactate (mmol/L)	1.9 (1.3, 2.9)	1.7 (1.1, 3.2)	1.9 (1.3, 2.9)	0.74
International Normalized Ratio	1.1 (1, 1.2)	1.2 (1.1, 1.2)	1.1 (1, 1.2)	0.076
Prothrombin time (s)	14.3 (13.6, 15.1)	14.7 (14.1, 15.4)	14.3 (13.6, 15.1)	0.065
Activated partial thromboplastin time (s)	36.7 (34.2, 39.8)	38 (35, 39.9)	36.7 (34.2, 39.8)	0.492
D-dimer (µg/L)	3.4 (1.4, 8.4)	5 (1.7, 15.9)	3.4 (1.4, 8.5)	0.074
Thrombocytocrit (%)	0.2 ± 0.1	0.2 ± 0.1	0.2 ± 0.1	0.962
Serum sodium (mmol/L)	140.7 ± 3.3	140.3 ± 3.6	140.7 ± 3.3	0.547
Serum calcium (mmol/L)	2.1 ± 0.1	2 ± 0.2	2.1 ± 0.1	0.495
Serum albumin (g/L)	34.1 ± 4.2	32.5 ± 4.5	34.1 ± 4.2	0.071
Serum creatinine (µmol/L)	58 (46, 69)	60 (50.8, 81.8)	58 (47, 70)	0.167
Serum total bilirubin (µmol/L)	13.6 (10.3, 18.3)	16.3 (10.6, 18.1)	13.7 (10.3, 18.3)	0.5
Serum urea (mmol/L)	4.8 (3.6, 6.2)	5.2 (4.1, 6.5)	4.8 (3.7, 6.2)	0.103
Alanine aminotransferase (U/L)	16 (12, 24)	15.5 (10.8, 22.5)	16 (12, 24)	0.46
Aspartate aminotransferase (U/L)	25 (20, 35)	27 (23.2, 46)	25 (20, 35)	0.096
**Outcome**				
Surgery time (hours)	3.2 ± 1.3	3.2 ± 1.3	3.2 ± 1.3	0.956
ICU length of stay (days)	4.7 (2.2, 10.5)	11.1 (3.7, 21.5)	4.7 (2.2, 10.6)	0.003
Mechanical ventilation time (days)	1.7 (0.7, 5.6)	9.2 (3, 13.7)	1.8 (0.7, 5.9)	<0.001
Hospital length of stay (days)	19 (15, 25)	26 (12.8, 40)	19 (15, 25)	0.095

**Note:**

Continuous variables are described by means and quarterbacks. Categories variables are analyzed by $\chi^2$ test and continuous variables are analyzed by Wilcoxon rank sum test. APACHE, Acute physiology and chronic health evaluation; ICU, intensive care unit; CKD, chronic kidney disease; SOFA, sepsis-related organ failure assessment.

### Univariate and multivariate logistic regression analyses

Table 3 displays the findings from both univariate and multivariate logistic regression analyses. In the univariate analysis, except for sex, neutrophil count, white blood cell count, serum urea, and serum sodium levels, all other variables in the table showed significant correlations with thrombocytopenia. In the multivariate analysis, factors that still had significant relationships with thrombocytopenia were SOFA score, hypertension, APACHE-II score, INR, thrombocytocrit, serum lactate level, serum total bilirubin level, and prothrombin time.

**Table 3:** Univariate and multivariate logistic regression analyses.

Variables	Univariate analysis		Multivariate analysis	
	OR [95% CI]	*P*-value	OR [95% CI]	*P*-value
Age (years)	1.021 [1.007–1.036]	0.005	1.012 [0.990–1.036]	0.288
Male	1.078 [0.712–1.654]	0.724		
Hypertension	0.336 [0.207–0.529]	<0.001	0.497 [0.261–0.923]	0.029
APACHE-II	1.141 [1.101–1.184]	<0.001	1.080 [1.022–1.143]	0.007
SOFA score	1.514 [1.386–1.660]	<0.001	1.175 [1.030–1.341]	0.016
Vasoactive drug used	1.250 [1.137–1.374]	<0.001	1.136 [0.979–1.308]	0.083
White blood cell count (×10^9^/L)	0.974 [0.920–1.030]	0.364		
Neutrophil count (×10^9^/L)	0.994 [0.936–1.054]	0.836		
Lymphocyte count (×10^9^/L)	0.461 [0.295–0.701]	<0.001	0.815 [0.466–1.396]	0.463
Red blood cell count (×10^12^/L)	0.274 [0.186–0.399]	<0.001	1.210 [0.703–2.085]	0.492
Serum sodium (mmol/L)	1.005 [0.945–1.068]	0.874		
Serum lactate (mmol/L)	1.294 [1.109–1.504]	<0.001	1.257 [1.018–1.552]	0.032
International normalized ratio*	2.183 [1.839–2.604]	<0.001	11.967 [2.960–50.066]	<0.001
Prothrombin time (s)	2.095 [1.767–2.496]	<0.001	0.130 [0.032–0.512]	0.004
APTT (s)	1.084 [1.038–1.132]	<0.001	0.943 [0.880–1.008]	0.089
D-dimer (µg/L)	1.106 [1.071–1.141]	<0.001	1.021 [0.973–1.070]	0.401
Serum albumin (g/L)	0.840 [0.797–0.883]	<0.001	0.960 [0.893–031]	0.268
Serum urea (mmol/L)	1.018 [0.921–1.122]	0.718		
Thrombocytocrit* (%)	0.700 [0.654–0.746]	<0.001	0.736 [0.672–0.801]	<0.001
Seum total bilirubin (µmol/L)	1.105 [1.073–1.138]	<0.001	1.074 [1.036–1.114]	<0.001
First measured systolic blood pressure (mmHg)	0.983 [0.976–0.990]	<0.001	0.992 [0.982–1.001]	0.098
Alanine aminotransferase (U/L)	1.023 [1.003–1.043]	0.021	1.035 [0.999–1.073]	0.054
Aspartate aminotransferase (U/L)	1.033 [1.018–1.048]	<0.001	0.978 [0.950–1.006]	0.130

**Notes:**

APACHE, Acute physiology and chronic health evaluation; SOFA, sepsis-related organ failure assessment; APTT, activated patial thromboplastin time.

* The OR and 95% CI for the international normalized ratio (INR) are calculated based on the INR values multiplied by 10. The OR and 95% CI for thrombocytocrit are calculated based on the thombocytocrit values multiplied by 100.

### Evaluation of logistic regression model

**Figure 4:**
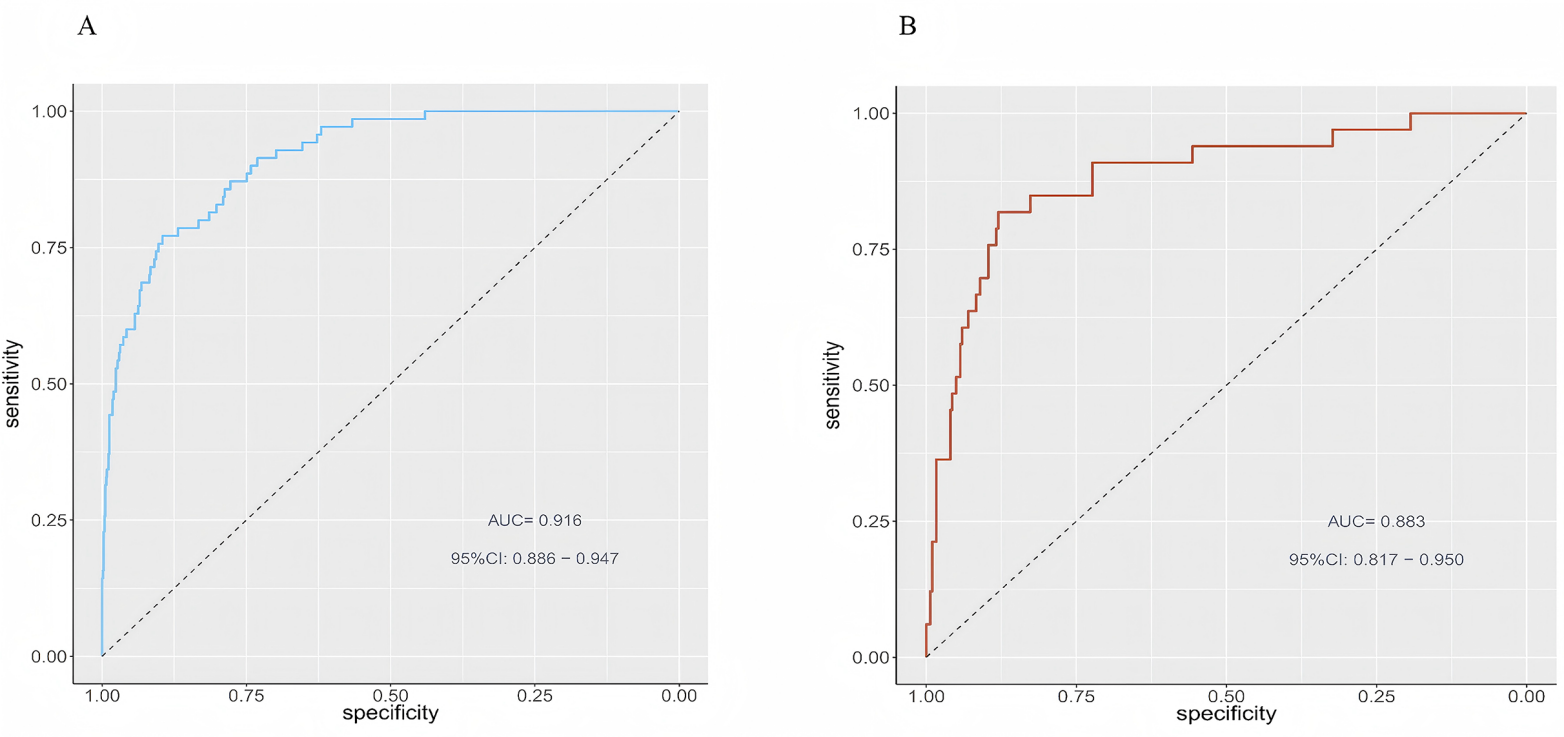
Receiver operating characteristic (ROC) curves of the prediction model for the training (A) and test (B) sets.

**Figure 5:**
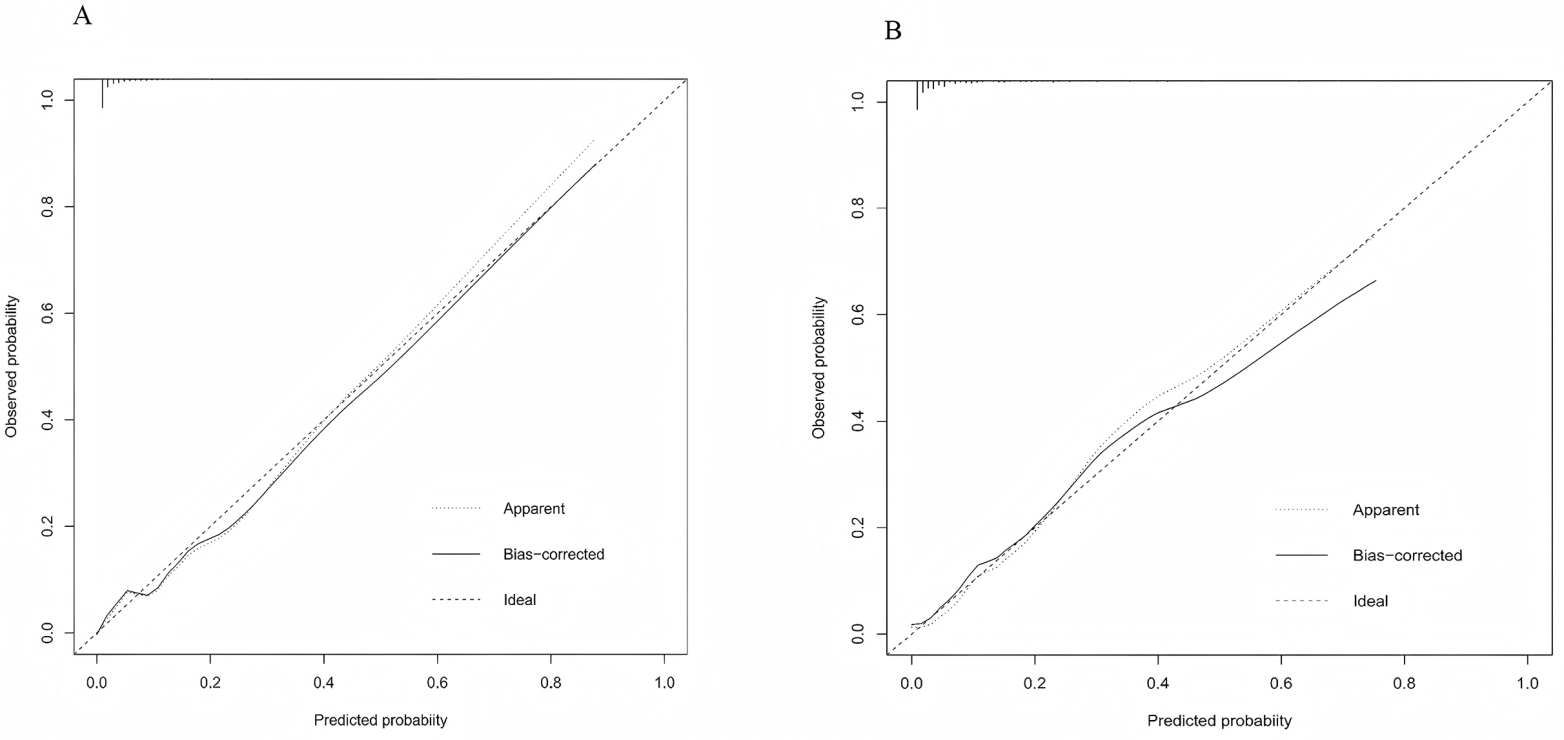
Calibration curves of the model for the training (A) and test (B) sets. The plots show the agreement between the predicted probabilities of thrombocytopenia and the observed outcomes. The dashed line represents the ideal fit (perfect calibration), while the solid line represents the model’s performance.

**Figure 6:**
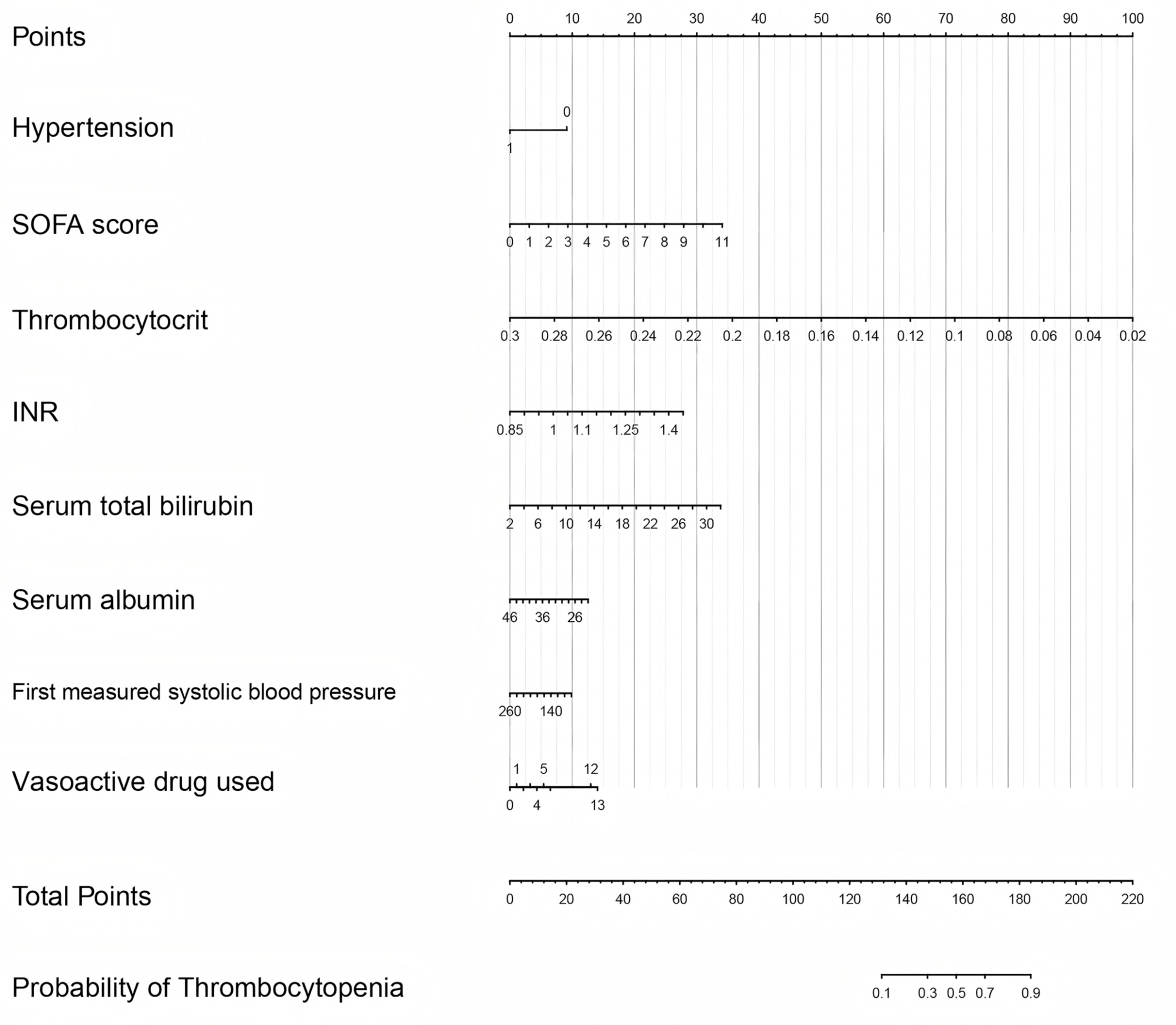
Nomogram for predicting the risk of postoperative thrombocytopenia. SOFA, Sepsis-related organ failure assessment; INR, international normalized ratio.

Figure 4 displays the ROC curves of the predictive model on the training and test datasets. The model performed well on both the training and test sets. The model’s AUC for the training dataset was 0.916 (95% CI [0.886–0.947]), and for the test dataset it was 0.883 (95% CI [0.817–0.950]). Figure 5 illustrates the calibration curves for the model. In the training dataset, the alignment of predicted and actual probability values closely approximated the diagonal line, indicating a high level of predictive accuracy for the model. In the test dataset, the alignment between predicted and actual probabilities was somewhat less precise, yet still within an acceptable range. Figure 6 displays the model’s nomogram for the test set. The evaluation metrics of the model, including recall, specificity, accuracy, and precision, were calculated using the confusion matrix. Because most of the patients in our sample did not have thrombocytopenia, to solve the problem of sample imbalance, we also calculated the F1-score. According to clinical and previous literature, the cutoff value was set to 0.5, the model reached an accuracy of 0.935, a recall of 0.414, a precision of 0.763, a specificity of 0.987, and a F1-score of 0.537 on the training set; and an accuracy of 0.907, a recall of 0.364, a precision of 0.545, a specificity of 0.967 and a F1-score of 0.436 on the test set. (Table 4).

**Table 4:** Model evaluation metrics on the training and test sets.

	Accuracy	Recall	Specificity	Precision	F1-score
Training set	0.935	0.414	0.987	0.763	0.537
Test set	0.907	0.364	0.967	0.545	0.436

### Model performance in etiology-specific subgroups

**Figure 7:**
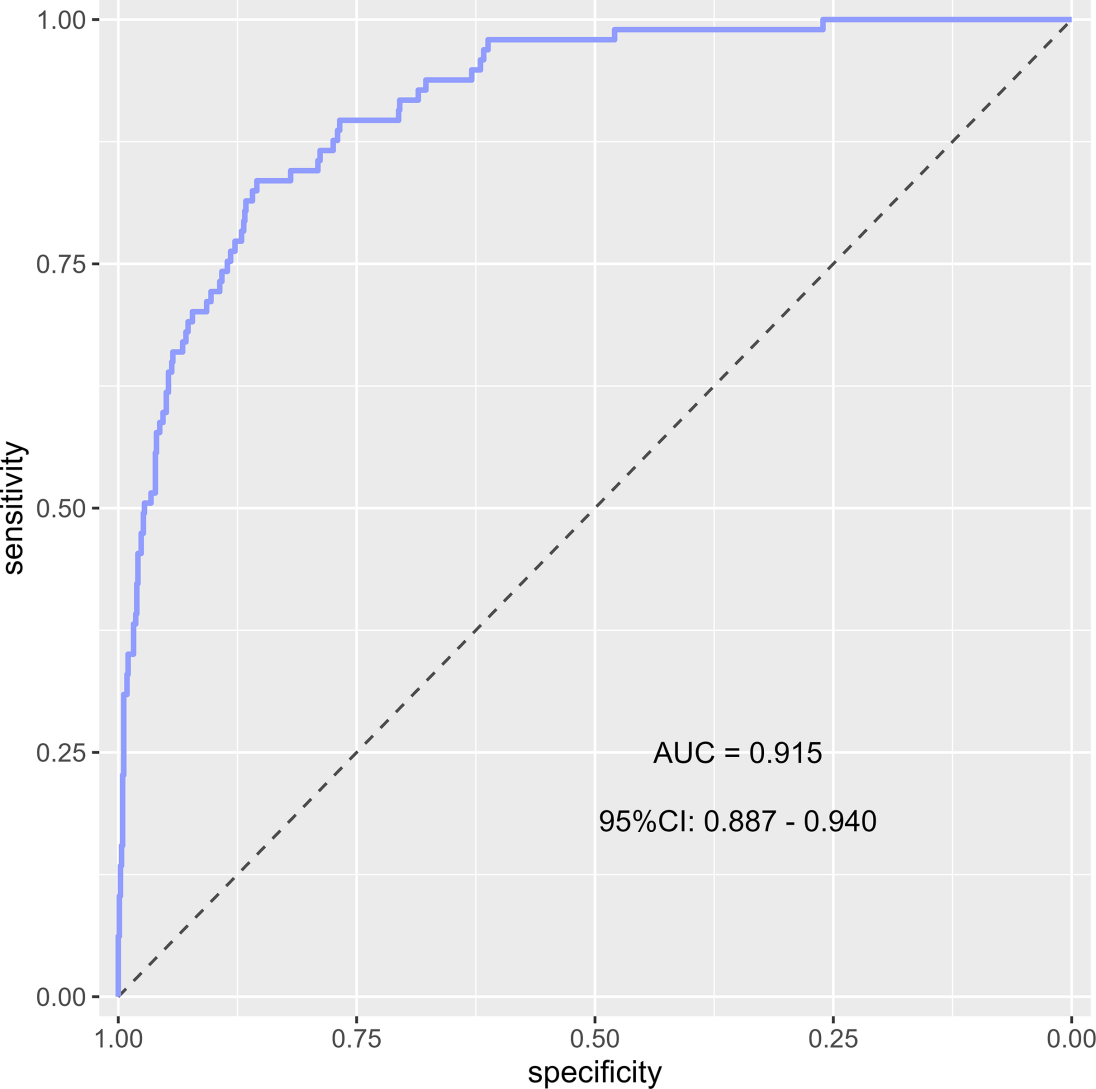
Receiver operating characteristic (ROC) curve of the prediction model for the acute injury subgroup. The apparent area under the curve (AUC) is 0.915 (95% CI [0.887–0.940]). The optimism-corrected AUC based on 1,000 bootstrap resamplings is 0.905 (95% CI [0.863–0.944]).

The predictor variables selected by LASSO regression for the acute injury group were SOFA score, APACHE-II score, INR, thrombocytocrit, serum total bilirubin level, and vasoactive drug use. For patients in the acute injury group, the model achieved an apparent AUC of 0.915 (95% CI [0.887–0.940]) (Fig. 7). The optimism-corrected AUC after 1,000 bootstrap resamplings remained robust at 0.905 (95% CI [0.863–0.944]). At a cutoff value of 0.5, the recall, specificity, accuracy, precision, and F1-score were 0.400, 0.978, 0.920, 0.664, and 0.499, respectively. A detailed comparison between the total population model and the acute injury subgroup model is presented in Table 5. In the elective surgery subgroup, the primary model correctly predicted 108 patients as non-thrombocytopenic. The specificity was 100%, and the NPV was 99.1%.

**Table 5:** Comparison of the total population model and the acute injury subgroup model.

Feature/Metric	Total population model	Acute injury subgroup model
Cohort focus	All neurosurgical patients	TBI and ICH patients only
Variable selection	LASSO regression	LASSO regression
Predictors	Hypertension, SOFA, Thrombocytocrit, INR, Total bilirubin, Albumin, First SBP, Vasoactive drugs	SOFA, APACHE-II, INR, Thrombocytocrit, Total bilirubin, Vasoactive drugs
Validation method	7:3 Data splitting (Train/Test)	1,000 Bootstrap resamplings
AUC (95% CI)	0.883 [0.817–0.950]	0.905 [0.863–0.944]
Recall	0.364	0.400
Specificity	0.967	0.978
Precision	0.545	0.664
Accuracy	0.907	0.920
F1-score	0.436	0.499

**Note:**

TBI, Traumatic brain injury; ICH, intracranial hemorrhage; SOFA, sepsis-related organ failure assessment; INR, international normalized ratio; APACHE-II, acute physiology and chronic health evaluation II; SBP, systolic blood pressure.

## Discussion

In our study, thrombocytopenia occurred in approximately 10% of the patients after neurosurgery. Another study by Xu et al. (2023) observed an incidence rate of thrombocytopenia at approximately 22% among patients who had undergone surgery for traumatic brain injury. One of the main reasons for this discrepancy is the different definitions of thrombocytopenia used. In the study by Xu et al. (2023), thrombocytopenia was defined as a platelet count below 150 × 10^9^/L. We did not perform a sensitivity analysis for different thrombocytopenia definitions. Consequently, we cannot definitively assert the robustness and generalizability of the model’s performance across the full spectrum of thrombocytopenia severity. Future research should conduct such sensitivity analyses, which would not only strengthen the model’s validity but also allow for the development of severity-specific risk scores.

In this study, our model exhibits an excellent AUC on the test set. For a binary classification model, a high AUC signifies strong overall discriminative power—the ability to correctly rank patients by risk, suggesting that the underlying risk architecture is correctly identified. When the cutoff value is set to 0.5, the model demonstrates high specificity and accuracy but a relatively moderate recall. This pattern indicates while the model is good at confirming the absence of thrombocytopenia, it may not be as effective in detecting all positive throbocytopenia cases. In clinical practice, the current performance of our model is perhaps best suited for application as a low-risk exclusion tool, rather than a highly sensitive diagnostic test. Clinicians can use the model to stratify patients predicted to be at low risk, allowing for a justified reduction in the frequency of routine platelet monitoring and the overall consumption of hematological resources for this cohort. Given the catastrophic consequences of missed thrombocytopenia in neurosurgery, clinical judgment remains necessary for managing potential false negatives. Overall, this logistic regression model demonstrated promise in predicting thrombocytopenia in patients after neurosurgery, but improving the model’s sensitivity remains a key objective for future work.

To address the clinical heterogeneity inherent in neurosurgical patients, we conducted a targeted subgroup analysis focusing on acute brain injuries (TBI and ICH). Our findings suggest that a disease-specific approach enhances the model’s predictive precision. Unlike the total population model, which incorporated long-term comorbidities (*e.g*., hypertension), the acute injury model prioritized markers of acute physiological distress—most notably the APACHE-II score. The inclusion of APACHE-II, alongside the SOFA score and vasoactive drug use, underscores the dominant role of systemic inflammatory response and acute multi-organ dysfunction in the pathogenesis of thrombocytopenia in the TBI and ICH patients. In these acute settings, the rapid consumption of platelets is often a manifestation of severe metabolic and physiological derangement, which is more accurately captured by comprehensive scores like APACHE-II than by pre-existing comorbidities. The model for the acute injury group achieved a higher precision (0.664) compared to the total population model (0.545), demonstrating its superior reliability in identifying true-positive cases.

Our study’s contribution is highlighted by a comparison with the majority of available prediction models for postoperative thrombocytopenia, which primarily focus on patient populations with distinct pathophysiologies, such as the prediction model for thrombocytopenia following coronary artery bypass graft (CABG) surgery by Zhao et al. (2024), and the transcatheter aortic valve replacement (TAVR) model by Li et al. (2023). The primary mechanism driving thrombocytopenia following CABG is often related to hemodilution and platelet consumption induced by cardiopulmonary bypass (CPB), making CPB duration a critical predictor. Similarly, the TAVR model by Li et al. (2023) relies heavily on factors such as low body weight, poor cardiac function, and major vascular complications, reflecting mechanisms tied to procedural trauma, bleeding risk, and systemic hemodilution. In contrast, our work provides the first model, to our knowledge, explicitly designed for the neurosurgical population. By identifying and integrating parameters highly sensitive to the neurosurgical environment, our model offers a timely risk stratification tool.

Although our introduction highlighted the unique propensity for TF-mediated consumptive coagulopathy in neurosurgery, the general systemic mechanisms are universally applicable to our neurosurgical cohort. Pathophysiologically, the main reasons for postoperative thrombocytopenia include platelet dilution, reduced platelet production, and enhanced platelet destruction (Nagrebetsky et al., 2019). Within the first few days after surgery, a decrease in the platelet count is usually caused by hemodilution due to intravenous fluid administration and accelerated platelet consumption caused by hemostasis. It takes 3–4 days for thrombopoietin to respond to the platelet decrease and increase platelet production. Some postoperative complications, such as multi-organ dysfunction syndrome and sepsis, may lead to a rapid decrease in platelet levels, as they can lead to disseminated intravascular coagulation, which pathologically accelerates platelet consumption (Skeith et al., 2020). Furthermore, the use of drugs can cause immune-mediated platelet destruction and bone marrow suppression, which can decrease platelet production (Nagrebetsky et al., 2019).

The eight most relevant features selected by LASSO regression to participate in the model establishment were: hypertension, SOFA score, thrombocytocrit, INR, serum total bilirubin, albumin, first measured systolic blood pressure, and vasoactive drugs. Platelet function in hypertension has been extensively studied. Functional and morphological changes in platelets in patients with hypertension indicate that they are hyperactivated and have an increased size, mean platelet mass, and mean platelet volume. Blood pressure has a positive linear relationship with a predisposition to platelet aggregation (El Haouari & Rosado, 2009). The SOFA score, which assesses six major organ systems—including the respiratory, cardiovascular, renal, hepatic, coagulation, and neurological systems—was originally designed to quantitatively and objectively describe the progression of organ dysfunction in critically ill patients, rather than solely to predict outcome or measure injury severity of a single organ (Vincent et al., 1996). The inclusion of INR and the SOFA score in our model provides indirect evidence of the underlying consumptive process. These parameters serve as sensitive indicators of accelerated systemic consumption and secondary organ dysfunction, which are characteristic of the hemostatic derangements seen in neurosurgical patients (Skeith et al., 2020). Platelet, bilirubin, and albumin levels are closely associated with liver function (Oettl et al., 2013; Chauhan et al., 2016; Hamoud et al., 2018). Bilirubin can cause abnormalities in platelet morphology and function, and the degree of abnormality correlates directly with bilirubin concentration (Suvansri, Cheung & Sawitsky, 1969). Conversely, low albumin levels are associated with enhanced clot formation, platelet aggregation, and increased primary hemostasis (Paar et al., 2017).

This study has several limitations. First, its retrospective design may introduce bias due to unmeasured potential confounders. Given the extensive polypharmacy common in the ICU, we could not individually analyze every specific medication known to affect platelet counts, including frequently employed antiepileptics such as sodium valproate. Although we adjusted for critical pharmacological categories—including vasoactive drugs (*e.g*., dopamine, norepinephrine), antibiotics, hormonal drugs, and antiplatelet agents—the specific exclusion of valproate and other individual agents means their independent effects could not be fully isolated. Future prospective studies with strict medication protocols are needed to further refine these associations. Furthermore, while we have provided data on measurable complications like deep vein thrombosis in our baseline analysis, other dynamic events such as postoperative hemorrhage were not included as predictors. This was a deliberate methodological choice to avoid look-ahead bias and ensure the model’s clinical utility for early intervention, as these complications often manifest after the initial assessment window.

Second, other crucial intraoperative and postoperative variables, such as specific surgical techniques, operator experience, and quantified blood loss, were not exhaustively captured. The omission of these factors restricts our ability to provide a complete mechanistic explanation for the observed associations. While postoperative physiological scores likely reflect the cumulative burden of these intraoperative events, the direct incorporation of such detailed data in future prospective studies is therefore essential to validate and refine our model.

Third, our study suffers from a lack of external validation. Since the data for our research was derived from a single institution, we were only able to perform internal validation. Moving forward, a multi-center prospective study is warranted to confirm the generalizability and robustness of our model across diverse neurosurgical practices and patient populations.

Fourth, the incidence of thrombocytopenia in our cohort was 9.3%, which resulted in a class imbalance. Future studies could employ advanced sampling techniques such as Synthetic Minority Over-sampling Technique (SMOTE) or implement cost-sensitive learning to specifically improve the sensitivity of the model.

## Conclusion

In conclusion, this study provides a valuable predictive tool for thrombocytopenia after neurosurgery. Through the development of a logistic regression model, we demonstrated its potential to predict platelet decline. Crucially, our subgroup analysis highlights the model’s superior performance in acute injury settings, where it achieved enhanced precision and a bootstrap-corrected AUC of 0.905. With further validation and refinement, these models could serve as clinical decision-support tools, enabling early intervention and potentially reducing the associated morbidity and mortality.

## Supplemental Information

10.7717/peerj.21094/supp-1 Supplemental Information 1 Data dictionary. The data of 1,109 patients included in the analysis.

10.7717/peerj.21094/supp-2 Supplemental Information 2 Data of the 1,109 patients included in the study.

10.7717/peerj.21094/supp-3 Supplemental Information 3 Codebook.

10.7717/peerj.21094/supp-4 Supplemental Information 4 Summary of algorithms and models used in this study.

## Additional Information and Declarations

### Data Availability

The following information was supplied regarding data availability:

The raw data is available in the Supplemental Files.
