# Supplementary material for: A prediction model for thrombocytopenia after neurosurgery: a retrospective study
Source: PeerJ. 2026 Apr 17;14:e21094. doi: 10.7717/peerj.21094 (PMC13094557; doi:10.7717/peerj.21094)
Supplement: Supplemental Information 1 — The data of 1,109 patients included in the analysis. [file peerj-14-21094-s001.docx]

Numeric Variables

| Variable Name | Variable Description | Units |
| --- | --- | --- |
| Age | Age | years |
| ICUstay | ICU length of stay | days |
| HXJstay | Mechanical ventilation time | days |
| Hosp..LOS | hospital length of stay | days |
| Surgery_time | Operative time | hours |
| Cost | Hospitalization cost | **10,000 Chinese Yuan** |
| SOFA | Sepsis-related Organ Failure Assessment Score | Points |
| APACHEII | Acute Physiology and Chronic Health Evaluation II score | Points |
| plt_icu_fri | Platelet count on the first day of ICU admission | x10^9^/L |
| plt_befor_icu_7day_max | Maximum platelet count within the 7 days preceding ICU admission |  |
| plt_befor_icu_2year_max | Maximum platelet count recorded within the 2 years preceding the current hospitalization |  |
| plt_icu_7day_min | Minimum platelet count within the first 7 days of ICU stay |  |
| plt_icu_2day_min | Minimum platelet count within the first 2 days of ICU stay |  |
| WBC | White blood cell count |  |
| Nuetrophil | Neutrophil leucocyte count |  |
| Lymphocyte | Lymphocyte count |  |
| RBC | Red blood cell count | X10^12^/L |
| Hematocrit | Hematocrite | L/L |
| Hemoglobin | Hemoglobin level | g/L |
| PCT.PLT. | Thrombocytocrit | % |
| Crp | C-reactive protein level | mg/L |
| PCT | Procalcitonin level | **μg/L** |
| pH | pH | - |
| Pco2 | Arterial carbon dioxide partial pressure | mmHg |
| Po2 | Arterial oxygen partial pressure | mmHg |
| Bicarbonate | Serum bicarbonate level | mmol/L |
| SO2 | Oxygen saturation | % |
| Lactate | Serum lactate level | mmol/L |
| FIO2 | Fraction of inspired oxygen | % |
| PT | Prothrombin time | seconds |
| INR | International Normalized Ratio | - |
| APTT | Activated Partial Thromboplastin Time | seconds |
| D.dimer | D-dimer level | µg/L |
| Ur | Serum urea level | mmol/L |
| Potassium | Serum potassium level |  |
| Sodium | Serum sodium level |  |
| Calcium | Serum calcium level |  |
| Magnesium | Serum Magnesium level |  |
| ALTicu | Alanine aminotransferase level in ICU | U/L |
| ASTicu | Aspartate aminotransferase level in ICU |  |
| Bilirubin_Total | Serum total bilirubin level | µmol/L |
| Bilirubin_Direct | Serum direct bilirubin level |  |
| Bilirubin_Indirect | Serum indirect bilirubin level |  |
| Albumin | Serum albumin level | g/L |
| Globulin | Serum globulin level |  |
| Palb | Plasma albumin level |  |
| glucose_fir | First blood glucose level upon ICU admission | mmol/L |
| glucose_min | Minimum blood glucose level within 24 hours upon ICU admission |  |
| glucose_max | Maximum blood glucose level within 24 hours upon ICU admission |  |
| glucose_mean | Mean blood glucose level within 24 hours upon ICU admission |  |
| sysbp_min | Minimum systolic blood pressure within 24 hours upon ICU admission | mmHg |
| sysbp_max | Maximum systolic blood pressure within 24 hours upon ICU admission |  |
| sysbp_mean | Mean systolic blood pressure within 24 hours upon ICU admission |  |
| sysbp_fir | First systolic blood pressure upon ICU admission |  |
| diasbp_fir | First diastolic blood pressure upon ICU admission |  |
| diasbp_min | Minimum diastolic blood pressure within 24 hours upon ICU admission |  |
| diasbp_max | Maximum diastolic blood pressure within 24 hours upon ICU admission |  |
| diasbp_mean | Mean diastolic blood pressure within 24 hours upon ICU admission |  |
| tempc_mean | Mean body temperature within 24 hours upon ICU admission | °C |
| tempc_min | Minimum body temperature within 24 hours upon ICU admission |  |
| tempc_max | Maximum body temperature within 24 hours upon ICU admission |  |
| tempc_fir | First body temperature upon ICU admission |  |
| spo2_fir | First peripheral oxygen saturation upon ICU admission | % |
| spo2_min | Minimum peripheral oxygen saturation within 24 hours upon ICU admission |  |
| spo2_max | Maximum peripheral oxygen saturation within 24 hours upon ICU admission |  |
| spo2_mean | Mean peripheral oxygen saturation within 24 hours upon ICU admission |  |
| heartrate_mean | Mean heart rate within 24 hours upon ICU admission | bmp |
| heartrate_min | Minimum heart rate within 24 hours upon ICU admission |  |
| heartrate_max | Maximum heart rate within 24 hours upon ICU admission |  |
| heartrate_fir | First heart rate upon ICU admission |  |
| resprate_max | Maximum respiratory rate within 24 hours upon ICU admission | RR |
| resprate_min | Minimum respiratory rate within 24 hours upon ICU admission |  |
| resprate_mean | Mean respiratory rate within 24 hours upon ICU admission |  |
| resprate_fri | First respiratory rate upon ICU admission |  |
| Cr_befor_icu_2year_min | Minimum creatinine level recorded within the 2 years preceding ICU admission | µmol/L |
| Cr_icu_7day_max | Maximum creatinine level within the first 7 days of ICU stay |  |
| Cr_icu_fri | First creatinine level upon ICU admission |  |
| gcs | Glasgow Coma Scale | points |

Binary or Categorical Variables

| Variable Name | Description | Value | Label/Encoding |
| --- | --- | --- | --- |
| Male | Gender | 0 | Female |
|  |  | 1 | Male |
| Hospital_mortality | Patient's vital status at the time of hospital discharge | 0 | Alive |
|  |  | 1 | Deceased |
| Smoking | Smoking status | 0 | No |
|  |  | 1 | Yes |
| Alcohol.drinking | Alcohol drinking habit | 0 | No |
|  |  | 1 | Yes |
| Hypertension | Hypertension | 0 | No |
|  |  | 1 | Yes |
| Diabete | Diabetes | 0 | No |
|  |  | 1 | Yes |
| CKD | Chronic Kidney Disease | 0 | No |
|  |  | 1 | Yes |
| myocardial.infarction | Myocardial Infarction | 0 | No |
|  |  | 1 | Yes |
| congestive.heart.failure | Congestive Heart Failure | 0 | No |
|  |  | 1 | Yes |
| Chronic.obstructive.pulmonary.disease | Chronic Obstructive Pulmonary Disease | 0 | No |
|  |  | 1 | Yes |
| Sepsis | Sepsis | 0 | No |
|  |  | 1 | Yes |
| Dopamine | Use of dopamine | 0 | No |
|  |  | 1 | Yes |
| CRRT | Continuous Renal Replacement Therapy | 0 | No |
|  |  | 1 | Yes |
| Dobutamine | Use of dobutamine | 0 | No |
|  |  | 1 | Yes |
| Milinong | Use of milrinong | 0 | No |
|  |  | 1 | Yes |
| Norepinephrine | Use of Norepinephrine | 0 | No |
|  |  | 1 | Yes |
| Adrenaline | Use of adrenaline | 0 | No |
|  |  | 1 | Yes |
| Posterior_pituitary | Use of posterior pituitary drugs | 0 | No |
|  |  | 1 | Yes |
| antibiotic_day1 | Use of antibiotics on admission day | 0 | No |
|  |  | 1 | Yes |
| antiplatelet_day1 | Use of antiplatelet drugs on admission day | 0 | No |
|  |  | 1 | Yes |
| hormone_day1 | Use of hormonal drugs on admission day | 0 | No |
|  |  | 1 | Yes |
| immunoglobulin_day1 | Use of immunoglobulin on admission day | 0 | No |
|  |  | 1 | Yes |
| AKI | Acute Kidney Injury | 0 | No |
|  |  | 1 | Yes |
| TP | Thrombocytopenia | 0 | No |
|  |  | 1 | Yes |
| shock_day1 | Use of vasoactive drugs on admission day | 0 | No |
|  |  | 1 | Yes |
| diagnosis | Preoperative diagnosis | 1 | Intracranial hemorrahge |
|  |  | 2 | Traumatic brain injury |
|  |  | 3 | Intracranial tumors |
|  |  | 4 | Unruptured cerebrovascular diseases |
|  |  | 5 | Other |
| op_site | Surgical site | 1 | Supratentorial |
|  |  | 2 | Infratentorial |
|  |  | 3 | Skull base |
|  |  | 4 | Other |
