# Supplementary material for: A prediction model for thrombocytopenia after neurosurgery: a retrospective study
Source: PeerJ. 2026 Apr 17;14:e21094. doi: 10.7717/peerj.21094 (PMC13094557; doi:10.7717/peerj.21094)
Supplement: Supplemental Information 3 [file peerj-14-21094-s003.docx]

Binary or categorical variables

| Variable Name | Description | Value | Label/Encoding |
| --- | --- | --- | --- |
| Male | Gender | 0 | Female |
|  |  | 1 | Male |
| Hospital_mortality | Patient's vital status at the time of hospital discharge | 0 | Alive |
|  |  | 1 | Deceased |
| Smoking | Smoking status | 0 | No |
|  |  | 1 | Yes |
| Alcohol.drinking | Alcohol drinking habit | 0 | No |
|  |  | 1 | Yes |
| Hypertension | Hypertension | 0 | No |
|  |  | 1 | Yes |
| Diabete | Diabetes | 0 | No |
|  |  | 1 | Yes |
| CKD | Chronic Kidney Disease | 0 | No |
|  |  | 1 | Yes |
| myocardial.infarction | Myocardial Infarction | 0 | No |
|  |  | 1 | Yes |
| congestive.heart.failure | Congestive Heart Failure | 0 | No |
|  |  | 1 | Yes |
| Chronic.obstructive.pulmonary.disease | Chronic Obstructive Pulmonary Disease | 0 | No |
|  |  | 1 | Yes |
| Sepsis | Sepsis | 0 | No |
|  |  | 1 | Yes |
| Dopamine | Use of dopamine | 0 | No |
|  |  | 1 | Yes |
| CRRT | Continuous Renal Replacement Therapy | 0 | No |
|  |  | 1 | Yes |
| Dobutamine | Use of dobutamine | 0 | No |
|  |  | 1 | Yes |
| Milinong | Use of milrinong | 0 | No |
|  |  | 1 | Yes |
| Norepinephrine | Use of Norepinephrine | 0 | No |
|  |  | 1 | Yes |
| Adrenaline | Use of adrenaline | 0 | No |
|  |  | 1 | Yes |
| Posterior_pituitary | Use of posterior pituitary drugs | 0 | No |
|  |  | 1 | Yes |
| antibiotic_day1 | Use of antibiotics on admission day | 0 | No |
|  |  | 1 | Yes |
| antiplatelet_day1 | Use of antiplatelet drugs on admission day | 0 | No |
|  |  | 1 | Yes |
| hormone_day1 | Use of hormonal drugs on admission day | 0 | No |
|  |  | 1 | Yes |
| immunoglobulin_day1 | Use of immunoglobulin on admission day | 0 | No |
|  |  | 1 | Yes |
| AKI | Acute Kidney Injury | 0 | No |
|  |  | 1 | Yes |
| TP | Thrombocytopenia | 0 | No |
|  |  | 1 | Yes |
| shock_day1 | Use of vasoactive drugs on admission day | 0 | No |
|  |  | 1 | Yes |
| diagnosis | Preoperative diagnosis | 1 | Intracranial hemorrahge |
|  |  | 2 | Traumatic brain injury |
|  |  | 3 | Intracranial tumors |
|  |  | 4 | Unruptured cerebrovascular diseases |
|  |  | 5 | Other |
| op_site | Surgical site | 1 | Supratentorial |
|  |  | 2 | Infratentorial |
|  |  | 3 | Skull base |
|  |  | 4 | Other |
