# Supplementary material for: A prediction model for thrombocytopenia after neurosurgery: a retrospective study
Source: PeerJ. 2026 Apr 17;14:e21094. doi: 10.7717/peerj.21094 (PMC13094557; doi:10.7717/peerj.21094)
Supplement: Supplemental Information 4 [file peerj-14-21094-s004.docx]

Supplemental Table 1 Summary of algorithms and models used in this study

| Stage | Algorithm / Model Used | Purpose | Software / R Package Used |
| --- | --- | --- | --- |
| Feature Selection | LASSO (Least Absolute Shrinkage and Selection Operator) Regression | To select the most relevant predictive variables | R (*glmnet* package) |
| Predictive Model | Logistic Regression | To build the final prediction model for postoperative thrombocytopenia | R (*stats* package, glm function) |
